# Impact of sarcopenia and obesity on mortality in older adults with SARS-CoV-2 infection: automated deep learning body composition analysis in the NAPKON-SUEP cohort

**DOI:** 10.1007/s15010-025-02555-3

**Published:** 2025-05-16

**Authors:** Sabine Schluessel, Benedikt Mueller, Olivia Tausendfreund, Michaela Rippl, Linda Deissler, Sebastian Martini, Ralf Schmidmaier, Sophia Stoecklein, Michael Ingrisch, Sabine Blaschke, Gunnar Brandhorst, Peter Spieth, Kristin Lehnert, Peter Heuschmann, Susana M. Nunes de Miranda, Michael Drey

**Affiliations:** 1 Department of Medicine IV, LMU University Hospital, LMU Munich, Munich, Germany; 2 Department of Radiology, LMU University Hospital, LMU Munich, Munich, Germany; 3 Department of Radiology, Clinical Data Science, LMU University Hospital, LMU Munich, Munich, Germany; 4 Emergency Department, University Medical Center Goettingen, Göttingen, Germany; 5 University Medicine Oldenburg, University Institute for Clinical Chemistry and Laboratory Medicine, Oldenburg, Germany; 6 Department of Anesthesiology and Intensive Care Medicine, Faculty of Medicine, University Hospital Carl Gustav Carus, TUD Dresden University of Technology, Dresden, Germany; 7 Department of Internal Medicine B, University Medicine Greifswald, Greifswald, Germany; 8 DZHK (German Center for Cardiovascular Research), University Medicine Greifswald, Greifswald, Germany; 9 Institute of Medical Data Science, University Hospital Würzburg, Würzburg, Germany; 10 Institute of Clinical Epidemiology and Biometry, University of Würzburg, Würzburg, Germany; 11 Faculty of Medicine, Institute for Digital Medicine and Clinical Data Science, Goethe University Frankfurt, Frankfurt, Germany; 12 Department I for Internal Medicine, Faculty of Medicine, University Hospital Cologne, University of Cologne, Cologne, Germany

**Keywords:** Geriatric patient, Body composition, Machine learning, Muscle assessment, Computed tomography, Muscle fat infiltration, Covid-19, Pneumonia

## Abstract

**Introduction:**

Severe respiratory infections pose a major challenge in clinical practice, especially in older adults. Body composition analysis could play a crucial role in risk assessment and therapeutic decision-making. This study investigates whether obesity or sarcopenia has a greater impact on mortality in patients with severe respiratory infections. The study focuses on the National Pandemic Cohort Network (NAPKON-SUEP) cohort, which includes patients over 60 years of age with confirmed severe COVID-19 pneumonia. An innovative approach was adopted, using pre-trained deep learning models for automated analysis of body composition based on routine thoracic CT scans.

**Methods:**

The study included 157 hospitalized patients (mean age 70 ± 8 years, 41% women, mortality rate 39%) from the NAPKON-SUEP cohort at 57 study sites. A pre-trained deep learning model was used to analyze body composition (muscle, bone, fat, and intramuscular fat volumes) from thoracic CT images of the NAPKON-SUEP cohort. Binary logistic regression was performed to investigate the association between obesity, sarcopenia, and mortality.

**Results:**

Non-survivors exhibited lower muscle volume (*p* = 0.043), higher intramuscular fat volume (*p* = 0.041), and a higher BMI (*p* = 0.031) compared to survivors. Among all body composition parameters, muscle volume adjusted to weight was the strongest predictor of mortality in the logistic regression model, even after adjusting for factors such as sex, age, diabetes, chronic lung disease and chronic kidney disease, (odds ratio = 0.516). In contrast, BMI did not show significant differences after adjustment for comorbidities.

**Conclusion:**

This study identifies muscle volume derived from routine CT scans as a major predictor of survival in patients with severe respiratory infections. The results underscore the potential of AI supported CT-based body composition analysis for risk stratification and clinical decision making, not only for COVID-19 patients but also for all patients over 60 years of age with severe acute respiratory infections. The innovative application of pre-trained deep learning models opens up new possibilities for automated and standardized assessment in clinical practice.

## Background

### Muscle loss as an underestimated factor in infectious medicine

Severe respiratory infections play a central role in clinical practice, especially in the aging population. They do not only pose an acute threat to survival but also have long term consequences. In recent years, there has been growing awareness that body composition – especially low muscle mass – plays a central role in the course and prognosis of infections [1, 2]. Sarcopenia, the age-related loss of muscle mass and strength, often remains unaddressed in clinical practice [3]. While fever, inflammatory markers and organ function are routinely monitored, muscle weakness is often overlooked as a potentially critical marker of disease progression [3].

### COVID-19 as a model for muscle weakness in infections

Coronavirus disease (COVID-19) has highlighted that infections not only affect organs but also body composition [4]. Numerous studies show that low muscle mass is associated with a poorer prognosis, longer ventilation times and higher mortality [4, 5]. The review by Wang et al. postulates that the link between sarcopenia and adverse COVID-19 outcomes is based on chronic inflammation, immune dysfunction and respiratory muscle weakness [4]. However, it remains unclear whether this is a specific phenomenon of COVID-19 or a general characteristic of severe infections. Current findings reveal strong parallels to sepsis, influenza and bacterial pneumonia [2, 6–8]. In sepsis patients, the massive inflammatory response leads to a catabolic metabolic state that contributes to rapid muscle atrophy [1, 2]. ICU-acquired weakness (ICUAW) is a well-known syndrome in intensive care patients that significantly impairs functional recovery after infections [1]. In acute respiratory distress syndrome (ARDS), too, there are indications that severely ill patients develop pronounced muscle weakness that can persist for years [9, 10]. These observations raise the question of whether muscle weakness should be considered as an independent risk factor for severe courses of infections and whether its early detection could enable targeted therapeutic intervention.

### Muscle tissue as an immunological player

Besides its mechanical function, the musculature also has a direct immunological significance. Muscle tissue produces myokines, which have inflammation-regulating properties and influence the systemic immune response [11, 12]. Reduced muscle mass could thus not only be a passive marker for a severe course of infection, but could actively contribute to the dysregulation of the immune system [13]. Patients with low muscle mass may have a weakened immune system, making them more susceptible to secondary infections and prolonged illness [13]. In addition, the musculature plays a crucial role in the storage of essential micronutrients, including vitamin D and iron, which are necessary for an effective immune response [14, 15]. A reduced muscle mass could thus indirectly lead to a weakened immune response [13].

### The impact of obesity and sarcopenia on health outcomes

While sarcopenia is increasingly recognized as a negative prognostic factor, it has been known for a long time that obesity is also associated with unfavorable disease progression, especially in critically ill patients [16]. Papadimitriou-Olivgeris et al. were able to show that obese patients with sepsis had a dramatically lower survival rate compared to patients of normal weight (survival rate 44% vs. 76%, *p* < 0.001) [17]. So far, it is unclear which nutritional condition – obesity or sarcopenia – is associated with a poorer prognosis [18]. Both conditions may interact in a complex way with inflammatory processes and the body’s immune response, which is why a differentiated analysis is necessary.

### Automated CT analysis as an innovative tool for infectious medicine

Determining body composition is challenging in the context of acute illnesses. Methods such as dual-energy X-ray absorptiometry (DXA) or bioelectrical impedance analysis (BIA) are hardly practical in acute medicine [19]. With the increasing use of deep learning technologies as part of machine learning, the automated analysis of CT scans offers a new way to quantify muscle volume in patients with infections precisely and in a standardized manner [20]. Thoracic CT scans are already part of routine imaging for severe infections such as COVID-19 or sepsis. The ability to automatically analyze body composition from these already existing image data opens up new avenues for risk stratification. Such an analysis could help infectious disease specialists to identify patients at increased risk early on and make targeted treatment decisions.

## Objective

Therefore, the main objective of this study was to investigate the effects of body composition on the survival rate of patients over 60 years of age with severe COVID-19 using a pre-trained deep learning model. In particular, it was analyzed whether obesity or sarcopenia was associated with a poorer prognosis. In addition, the extent to which these patient groups differed in terms of their comorbidities was investigated.

## Methods

### Study design and sample

The cross-sectoral platform (SUEP) cohort of the National Pandemic Cohort Network (NAPKON) recruited SARS-CoV-2-positive patients of all age groups from all sectors of the healthcare system in Germany (university hospitals, non-university hospitals and GP practices). Since November, 2020, the cohort has included both inpatients and outpatients. The SUEP cohort collects primary health data, basic clinical phenotyping information, imaging data, biospecimens and patient-reported outcome measures at 57 study sites. The main inclusion criterion was a polymerase chain reaction (PCR)-confirmed SARS-CoV-2 infection at the time of study participation. Detailed information on the study has been published elsewhere [21].

Our sample was recruited between November 1, 2020 and July 17, 2022. The dataset was exported on December 1, 2022. For the purposes of our research question, only patients aged > 60 years with thoracic CT imaging were included in our study. Only CT images obtained during the acute phase of hospitalization were included. The study protocol was approved by the LMU Munich Ethics Committee (study no. 22–0671).

### Data acquisition

The following NAPKON-SUEP variables were analyzed at baseline: age, gender, risk factors, blood pressure, SARS-CoV-2 vaccination status, life expectancy, restrictions on medical interventions, oxygen support, pre-existing conditions and time of CT imaging. Anthropometric data (height and weight) were collected at baseline through patient self-report during anamnesis, as part of the standardized NAPKON SUEP protocol. For each patient, a single axial chest CT image was selected and the position of the arms during the CT was documented.

In the NAPKON-SUEP cohort, patient mortality was documented through systematic recording of clinical outcomes during the acute phase. This information was recorded in electronic case report forms and verified through regular quality controls. In addition, follow-up visits were conducted in the SUEP cohort three and twelve months after the initial infection, supplemented by telephone interviews every six weeks. These follow-ups enabled continuous monitoring of the patients’ health status and contributed to the validation of the mortality data.

### Body composition analysis

The thoracic CT images were used for deep learning-based body composition analysis. Before the extraction of body composition markers, the CT images were resampled to a slice thickness of 5 mm to ensure a uniform database for further analysis. The body composition markers were then calculated using a fully automated evaluation pipeline. The analysis focused on the thorax, excluding the extremities. As described by Hosch et al., the thoracic cavity was defined as the chamber enclosed by the rib cage, extending from the superior thoracic aperture to the diaphragm, and includes the trachea up to the level of the cricoid cartilage [22]. For this region, the volumes of the relevant body structures were determined individually, allowing for the separate assessment of muscle mass, total fat mass, and intramuscular fat mass in alignment with our research objectives. All raw parameters were given as volumes (mL).

Further details about the Smart Hospital Information Platform (SHIP)-AI software of the University Hospital Essen have been documented elsewhere [23, 24].

### Clinical definitions

According to the WHO definition, obesity was defined as BMI ≥ 30 kg/m² [25]. Sarcopenia was assessed via normalized muscle volume (to weight and height²) based on CT data.

### Statistics

IBM^®^ SPSS^®^ Statistics version 29 was used for statistical analysis and R studio was utilized for graph design. Metric variables were presented as means and standard deviations (SDs), while categorical variables were presented as frequencies and percentages. Metric variables were compared between the two groups using Student’s t-test, while categorical variables were analyzed using the chi-square test or, if applicable, the exact Fisher test. The parameters from pretrained automated body composition analysis were all extracted as volumes. In order to follow current consensus statements for sarcopenia and sarcopenic obesity the muscle volumes were additionally normalized to weight and height squared [26, 27]. Furthermore, body composition parameters were visualized as density plots for the survivors and non-survivors groups.

Also, we conducted a binary logistic regression with death as the dependent variable. The metric independent variables were standardized to their SDs for comparability. Four regression models were formulated for the analysis. Each model considered either muscle volume or its normalized variants, total fat volume, intramuscular fat volume or BMI as an independent variable. Model 1 remained unadjusted, while model 2 was adjusted for age and sex. Model 3 was additionally adjusted for diabetes and chronic lung disease. Model 4 was additionally adjusted forchronic kidney disease. The results were expressed as odds ratios (OR) with confidence intervals and visualized in OR plots. A p-value < 0.05 was considered statistically significant in the entire analysis.

## Results

### Patient characteristics

A total of 157 patients (mean age: 70 ± 8 years; 41% women) met our inclusion criteria. The patient characteristics are shown in Table 1.

The patient collective corresponded to a geriatric cohort, since over 90% had at least two comorbidities and were thus classified as multimorbid. The average BMI was 28.4 kg/m^2^, with about a third of patients with a BMI > 30 kg/m^2^ considered obese.

All included patients suffered from severe COVID-19, as all required oxygen therapy.

Of the 157 patients, a total of 61 (39%) died. The groups death and control did not differ in terms of age, gender distribution and smoking status. However, the non-survivors had a significantly lower vaccination rate (16% vs. 55%), were more likely to have a medical intervention limitation (39% vs. 5%) and had a higher BMI (29.9 kg/m^2^ vs. 27.5 kg/m^2^) compared to survivors. In addition, diabetes, chronic lung disease and chronic kidney disease were significantly more common in the non-survivor group.

The majority of CT scans (78%) were performed as baseline examinations. Since arm position in the CT can influence the measurement of muscle mass, this was documented, but did not differ between the two groups It was found that the majority of patients (95%) stretched both arms above their heads, which meant that the imaging conditions were homogeneous.

**Table 1:** Patient characteristics

Characteristics	all	death	control	*p*-value
n (%)	157 (100.0)	61 (38.9)	96 (61.1)	
**Demographic variables**				
age [years]	70 (8)	71 (8)	69 (8)	0.107 ^a^
age groups [years]				
60–69 years	82 (52.2)	27 (44.3)	55 (57.3)	0.280^a^
70–79 years	49 (31.2)	22 (36.0)	27 (28.1)	
>80 years	26 (16.6)	12 (19.7)	14 (14.6)	
women (%)	65 (41)	24 (39)	41 (43)	0.677 ^a^
**Risk factors**				
BMI [kg/m^2^]	28.4 (6.0)	29.9 (7.3)	27.5 (5.0)	**0.031** ^b^
Obesity (BMI ≥ 30 kg/m²) (%)	38 (24.2)	17 (27.8)	21 (21.9)	0.165 ^b^
Ever smoker (%)	48 (30.6)	17 (27.9)	31 (32.3)	0.253 ^c^
**Blood pressure**				
Systolic [mmHg]	128 (20)	122 (22)	132 (19)	**0.004** ^d^
Diastolic [mmHg]	70 (14)	64 (14)	74 (13)	**< 0.001** ^d^
**Vaccination and life expectancy**				
SARS-CoV-2 vaccination (%)	63 (40.1)	10 (16.4)	53 (55.2)	**< 0.001** ^e^
Life expectancy of less than one year (%)	6 (3.7)	5 (8.2)	1 (1.0)	**0.020** ^f^
Limitation of medical interventions (%)	29 (18.5)	24 (39.3)	5 (5.2)	**< 0.001** ^g^
**Oxygenation support**				**< 0.001** ^h^
Conventional oxygen therapy (%)	57 (42.2)	9 (16.1)	48 (60.8)	
High-Flow oxygen therapy (%)	10 (7.4)	4 (7.1)	6 (7.6)	
Non-invasive mechanical ventilation (%)	24 (17.8)	8 (14.3)	16 (20.2)	
Invasive mechanical ventilation (%)	44 (32.6)	35 (62.5)	9 (11.4)	
**Disease**				
Multimorbidity (≥ 2) (%)	128 (89.5)	54 (94.7)	74 (86.0)	0.097 ^i^
High blood pressure (%)	107 (93.9)	46 (93.9)	61 (92.4)	1.000 ^j^
Heart disease (%)	117 (75.0)	50 (83.3)	67 (69.8)	0.057 ^k^
Diabetes mellitus (%)	56 (36.4)	28 (46.7)	28 (29.8)	**0.034** ^l^
Chronic lung disease (%)	47 (30.3)	24 (40.7)	23 (24.0)	**0.028** ^m^
Gastrointestinal disease (%)	10 (6.6)	6 (10.5)	4 (4.3)	0.179 ^n^
Neurological or psychiatric disease (%)	27 (17.4)	13 (21.7)	14 (14.7)	0.268 ^m^
Solid tumor disease (%)	32 (20.5)	14 (23.3)	18 (18.6)	0.490 ^k^
Hematological malignancy (%)	17 (11.1)	9 (15.0)	8 (8.6)	0.219 ^o^
Chronic renal disease (%)	32 (21.1)	22 (37.3)	10 (10.8)	**< 0.001** ^d^
Chronic liver disease (%)	12 (7.8)	6 (10.2)	6 (6.4)	0.396 ^o^
Rheumatologic condition (%)	8 (5.1)	3 (4.9)	5 (5.2)	0.936 ^a^
Post organ transplantation (%)	5 (3.2)	3 (5.0)	2 (2.1)	0.670 ^m^
**Arm positioning during CT scan**				
Both arms above the head (%)	142 (95.3)	53 (94.6)	89 (95.7)	0.634 ^r^
One arm above the head (%)	4 (2.5)	1 (1.8)	3 (3.2)	
Both arms alongside the body (%)	3 (1.9)	2 (3.6)	1 (1.1)	

BMI: body mass index. CT: computer tomography

^a^ Variable was available for 157 patients

^b^ Variable was available for 126 patients

^c^ Variable was available for 111 patients

^d^ Variable was available for 152 patients

^e^ Variable was available for 119 patients

^f^ Variable was available for 131 patients

^g^ Variable was available for 148 patients

^h^ Variable was available for 135 patients

^i^ Variable was available for 143 patients

^j^ Variable was available for 115 patients

^k^ Variable was available for 156 patients

^l^ Variable was available for 154 patients

^m^ Variable was available for 155 patients

^n^ Variable was available for 151 patients

^o^ Variable was available for 153 patients

^r^ Variable was available for 149 patients

### Body composition analysis of survivors and non-survivors

**Fig. 1:**
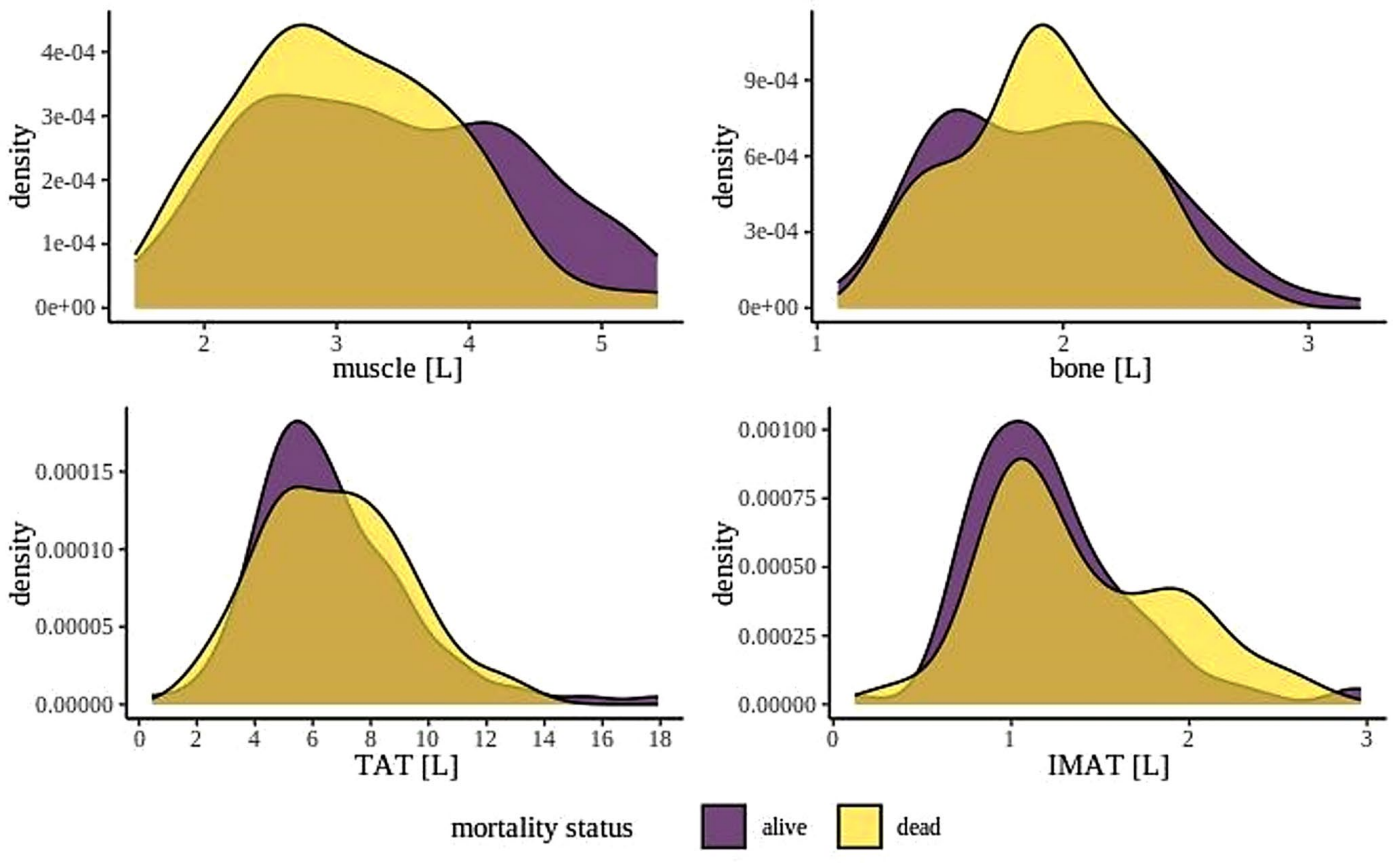
Density plot of body composition TAT = total adipose tissue, IMAT = intramuscular adipose tissue. The color scheme was specifically chosen so that it is easily distinguishable even for patients with red-green color deficiency

Thoracic CT examination was performed at a mean interval of six days following a positive SARS-CoV-2 swab test. Body composition analyses derived from thoracic CT scans without extremities are shown in Table 2. Non-survivors had significantly lower total muscle volume and higher intramuscular fat volume compared to survivors. This difference was particularly noticeable when muscle volume was considered in relation to body weight. In contrast, no significant differences were found between the two groups in terms of bone and total fat volume. Figure 1 indicates these results as density plots.

**Fig. 2:**
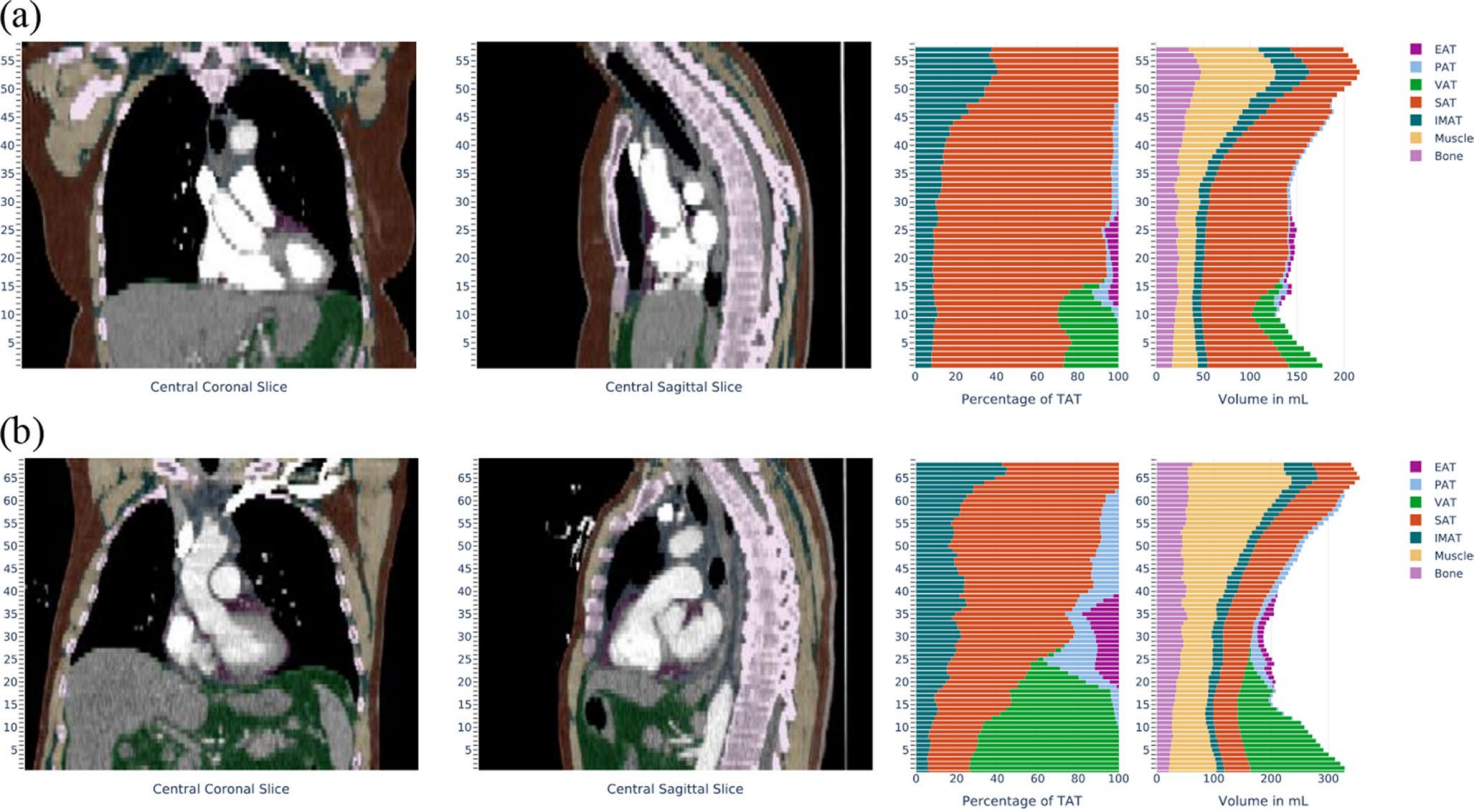
Reports of the body composition analysis of two examples: (**a**) with low muscle volume and (**b**) with sufficient muscle volume EAT = epicardial adipose tissue, PAT = pericardial adipose tissue, VAT = visceral adipose tissue, SAT = subcutaneous adipose tissue, IMAT = intramuscular adipose tissue

Figure 2 shows two examples of thoracic CT scans in coronal and sagittal view. Both subjects presented are in the typical scanning position with their arms raised above their heads. The automated analysis of the CT scan in example (a) indicates a lower muscle volume and a greater amount of subcutaneous adipose tissue (SAT) compared to the second case (b). It should be noted that Fig. 2 shows only a single section, whereas the automated body composition analysis took into account all 5-mm slices of the thoracic CT scan.

**Table 2:** Body composition analysis of thoracic CT scans

	all	death	control	*p*-value
n (%)	157 (100.0)	61 (38.9)	96 (61.1)	
Muscle volume [L]	3.26 (0.94)	3.07 (0.81)	3.38 (1.00)	**0.043** ^a^
Muscle volume/ height^2^ [L/m^2^]	1.10 (0.27)	1.05 (0.26)	1.13 (0.27)	0.111 ^p^
Muscle volume/ weight [L/kg]	0.0399 (0.01)	0.0364 (0.01)	0.0417 (0.01)	**0.003** ^q^
Bone volume [L]	1.95 (0.40)	1.93 (0.35)	1.96 (0.44)	0.736 ^a^
Total fat volume [L]	6.71 (2.62)	6.79 (2.48)	6.65 (2.72)	0.760 ^a^
Intramuscular fat volume [L]	1.28 (0.50)	1.39 (0.53)	1.22 (0.47)	**0.041** ^a^

^a^ Variable was available for 157 patients

^p^ Variable was available for 141 patients

^q^ Variable was available for 134 patients

### Influence factors on mortality

**Fig. 3:**
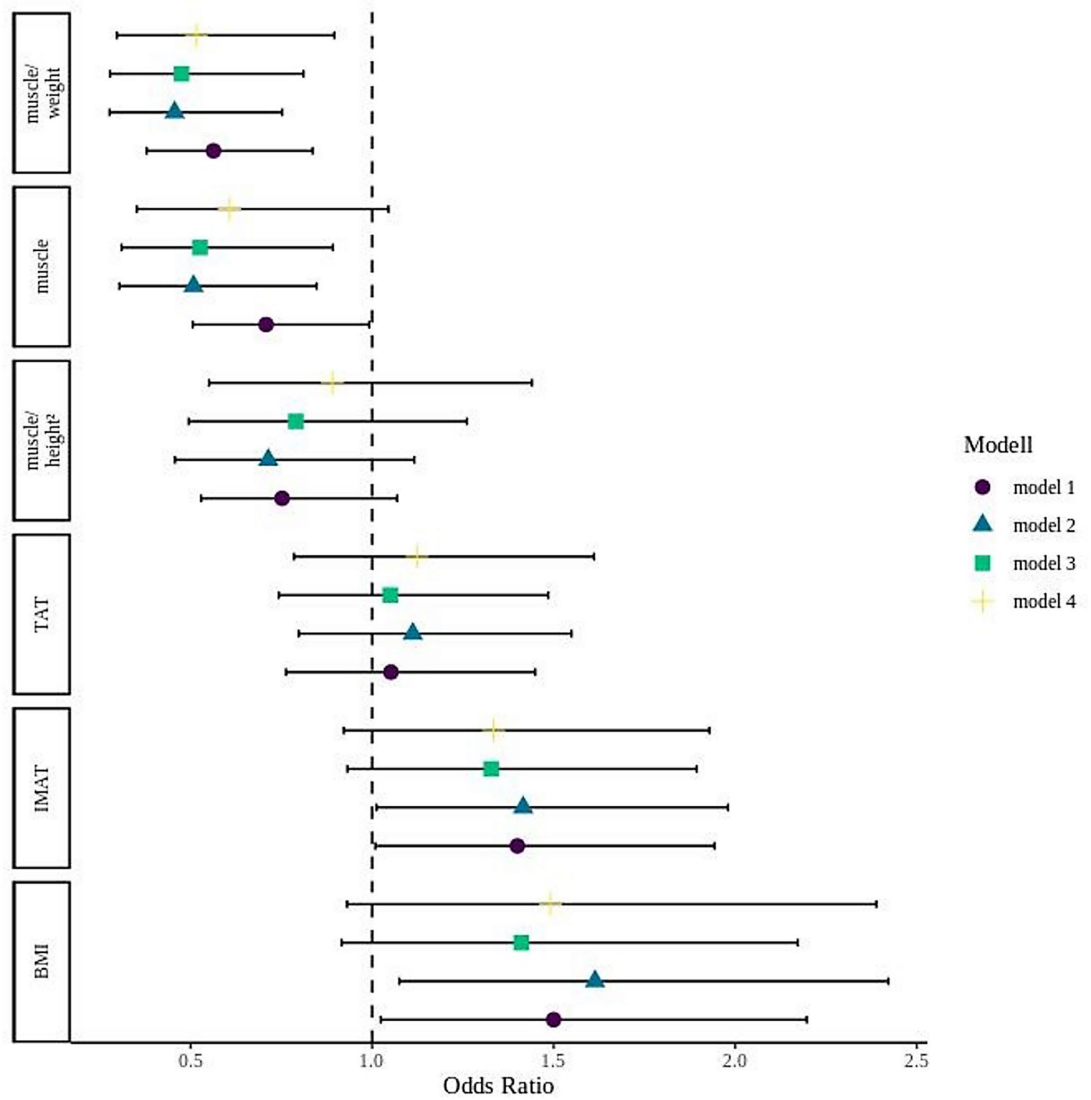
Odds ratio plot for the binary logistic regression BMI: body mass index, TAT = total adipose tissue, IMAT = intramuscular adipose tissue. Model 1: unadjusted. Model 2: adjusted for age and sex. Model 3: adjusted for age, sex, diabetes and chronic lung disease Model 4: adjusted for age, sex, diabetes, chronic lung disease. and chronic kidney disease

The results of the binary logistic regression analysis are presented in Table 3, with death as the dependent variable. Four models were evaluated. In the unadjusted model (Model 1) as well as in the model adjusted for age and gender (Model 2), muscle volume, muscle volume normalized to body weight, BMI, and intramuscular fat volume were significantly associated with mortality. In Model 3, which was further adjusted for diabetes and chronic lung disease, these associations remained largely consistent. However, in the fully adjusted model (Model 4), which also included chronic kidney disease, only muscle volume normalized to weight remained a significant predictor of mortality (OR 0.516, 95% CI 0.297–0.896, *p* < 0.01). Absolute muscle volume, BMI, and intramuscular fat volume no longer showed significant associations. These findings suggest that muscle volume relative to body weight may be a more robust and independent marker of mortality risk in this cohort. The results are further illustrated by the odds ratio plots in Fig. 3.

**Table 3:** Logistic regression analysis with death as dependent variable

Independent variables	model 1	model 2	model 3	model 4
Muscle volume	**0.708** **(0.506–0.992)**^a^ *	**0.508** **(0.304–0.847)** ^a^ **	**0.526** **(0.310–0.892)**^n^ *	0.607 (0.352–1.045) ^z^
Muscle volume/ weight	**0.563** **(0.379–0.836)** ^q^ **	**0.456** **(0.277–0.752)** ^q^ **	**0.475** **(0.278–0.811)** ^s^ **	**0.516** **(0.297–0.896)** ^t^ **
Muscle volume/ height^2^	0.752 (0.529–1.069) ^p^	0.714 (0.457–1.116) ^p^	0.790 (0.495–1.261) ^u^	0.891 (0.551–1.440) ^v^
Total fat volume	1.052 (0.763–1.449) ^a^	1.112 (0.798–1.549) ^a^	1.050 (0.743–1.485) ^n^	1.124 (0.785–1.611) ^z^
Intramuscular fat volume	**1.400** **(1.009–1.943)**^a^ *	**1.416** **(1.012–1.980)** ^a^ *	1.328 (0.932–1.894) ^n^	1.334 (0.922–1.929) ^z^
BMI	**1.500** **(1.024–2.197)** ^b^ *	**1.614** **(1.075–2.422)** ^b^ *	1.411 (0.916–2.172) ^e^	1.491 (0.931–2.386) ^w^

BMI: body mass index

Model 1 is unadjusted

Model 2 is adjusted for age and gender

Model 3 is adjusted for age, gender, diabetes and chronic lung disease

Model 4 is adjusted for age, gender, diabetes, chronic lung disease and chronic renal disease

Significance levels are expressed as * <0.05 and **<0.01

^a^ Variable was available for 157 patients

^b^ Variable was available for 126 patients

^e^ Variable was available for 122 patients

^n^ Variable was available for 153 patients

^p^ Variable was available for 141 patients

^q^ Variable was available for 134 patients

^s^ Variable was available for 130 patients

^t^ Variable was available for 128 patients

^u^ Variable was available for 137 patients

^v^ Variable was available for 135 patients

^w^ Variable was available for 135 patients

^z^ Variable was available for 151 patients

## Discussion

A total of 157 patients from the NAPKON-SUEP cohort (mean age: 70 ± 8 years; 41% women) were included in our study. To the best of our knowledge, we are the first to investigate muscle and fat volume using automated 3D-CT-Thorax analysis in a cohort of COVID-19 patients aged > 60 years. Our results showed that muscle mass was the most relevant body composition factor in relation to mortality.

### Low muscle mass as marker for severe courses of infection

Our regression analysis showed that muscle volume adjusted to weight had the strongest influence on survival, even after adjustment for age, sex, diabetes mellitus, chronic lung and kidney disease. This suggests that muscle volume adjusted to weight plays a central role in the prognosis of severe COVID-19 pneumonia. Additionally, we could show that for BMI and as well total muscle fat this effect did not remain significant after adjusting for comorbidities. Therefore, we postulate that sarcopenia plays a greater role than obesity in patients aging over 60 years with severe respiratory infection and that muscle volume is the most reliable body composition marker for predicting mortality.

Our findings align with previous studies, although most of these focused either on muscle or fat tissue and examined thoracic or abdominal CT scans in COVID-19 patients [5, 28–33]. Moreover, the vast majority relied on two-dimensional measurements of a single cross-sectional slice, typically assessing muscle structures at the thoracic vertebrae T4 and T12, the lumbar vertebrae L2 and L3 and the pectoralis muscle [5, 28–33]. Due to different anatomical reference points, the findings of these studies were sometimes inconsistent. A recent review including 22 studies on the association between sarcopenia and mortality in COVID-19 patients found that 17 studies reported a direct correlation, whereas five showed no significant association [5]. However, none of these studies employed a three-dimensional approach [5], which may have led to inconsistent findings.

Nevertheless, the results show that obesity and sarcopenia need to be considered carefully. When both conditions are present at the same time, this condition is referred to as sarcopenic obesity (SO), which is associated with particularly unfavorable health consequences. Long-term studies have shown that patients with SO have the poorest survival prognosis, followed by patients with sarcopenia alone and finally by obese patients without sarcopenia [34].

Another noteworthy finding is the significant difference in intramuscular fat content in our cohort. Non survivors showed significantly higher amounts of intramuscular fat. This observation is consistent with the long-term study by Pishgar et al., which showed that increased intermuscular fat was associated with higher mortality, while subcutaneous fat had less of an impact – a pattern also observed in patients with chronic obstructive pulmonary disease (COPD) [35]. In view of these results, intramuscular fat could be used as a potential prognostic marker in future studies and in clinical practice [36].

### Normalization of muscle volume

Another relevant aspect is the normalization of muscle volume. For decades, there has been discussion – similar to that regarding BMI – about which method of normalizing muscle volume is most useful. According to the definition of sarcopenia, normalization to height squared is recommended [26]. However, recent studies on sarcopenic obesity indicate that this method may lead to an overestimation of muscle mass in obese patients (BMI ≥ 30 kg/m^2^ and/or increased waist circumference). This is because obese patients often have greater muscle mass due to their higher body weight, but this is not necessarily associated with sufficient muscle strength and function [27]. For this reason, since 2022, normalization to body weight has been increasingly favored [27]. In our study, only muscle volume normalized to weight remained a significant predictor of mortality in the fully adjusted model, while absolute muscle volume and muscle volume normalized to height² lost statistical significance. This supports the notion that normalization to weight may be more appropriate in cohorts with a higher prevalence of overweight and obesity. Indeed, the average BMI in our cohort was 28 kg/m², compared to 25 kg/m² in our previous study [37], where no clear advantage was found for weight-based normalization. This difference might explain the shift in results and highlights the importance of tailoring normalization strategies to the specific characteristics of the population under study.

Interestingly, in both studies, absolute muscle volume showed strong correlations with normalized values, although its predictive value was not robust after full adjustment in the current analysis. This underscores the potential value of simple absolute measures, but also the importance of adjusting for body composition when assessing clinical risk.

### Additional factors influencing mortality beyond muscle mass

The analysis of the patient groups shows significant differences in several clinical and demographic factors that are potentially associated with mortality and are already well described in recent publications [38, 39]: The vaccination status differed significantly between the groups: in the group of non-survivors, it was only 16%, while in the survivors it was 55%. Additionally, life expectancy of less than one year and limitation of medical interventions was significant higher in the non-survivor group.

In term of comorbidities, the two groups differed significantly in terms of three concomitant diseases: diabetes mellitus, chronic renal failure and chronic lung disease. Recent major studies confirm this correlation and show that these comorbidities increase the mortality risk in the event of severe Covid-19-infections [40–42]. Interestingly, no significant differences were found with regard to cardiovascular diseases, including arterial hypertension. In addition, the mean blood pressure in the group of non-survivors was significantly lower. This could be explained by severe septic processes with consecutive blood pressure drop. However, this raises the question of whether diabetes, chronic renal insufficiency and lung diseases play a greater role than cardiovascular risk factors in severe respiratory infections but cannot be answered by this study design.

### Therapeutic consequences and clinical relevance

The realization that low muscle mass is associated with higher mortality highlights the importance of preventive and therapeutic measures to maintain muscle mass, especially in infectious medicine. It is therefore crucial to take measures that promote muscle maintenance – both preventively and therapeutically. Early detection of patients with lower muscle mass could help to counteract this. The use of automated methods as showed in our study from routine CT scans could prove to be a valuable tool for risk assessment. This would allow the early identification of patients with low muscle mass, who could then be monitored more closely [20]. In addition to diagnostic options, nutrition plays a central role [14, 15, 43]. A protein-rich diet combined with essential amino acids can help to slow down muscle breakdown and at the same time strengthen the immune system. Likewise, the targeted intake of micronutrients such as vitamin D and iron could have a positive influence on the course of the disease [14, 15]. Physiotherapy is also of great importance. Especially for seriously ill patients, it is essential to integrate early exercise programs to minimize muscle loss [44]. For ventilated patients in particular, respiratory muscle training programs could help to stabilize respiratory function and accelerate recovery [45]. Future studies should also focus on the automated analysis of diaphragm and respiratory muscle composition to gain better insight into respiratory sarcopenia [46].

### Automated body composition analysis in other medical contexts

The automated, AI-based analysis of body composition using CT imaging has gained increasing importance across various medical disciplines in recent years. Several studies have demonstrated a strong correlation between muscle and fat measurements derived from thoracic CT scans and those obtained from abdominal CT at the L3 vertebral level, which is considered the gold standard in body composition analysis [47]. The SHIP-AI system used in this study was first described 2021 by Koitka et al. and showed a high degree of agreement with established reference methods such as DXA and BIA – particularly with regard to fat distribution and muscle mass [23]. In a follow-up study by Kroll et al., SHIP-AI was successfully used for quantitative assessment in patients with neuroendocrine tumors [24]. In addition, it has recently been shown that SHIP-AI-based indices such as the sarcopenia or fat index also have prognostic relevance in patients with idiopathic pulmonary fibrosis [48]. These studies illustrate the broad spectrum of application and clinical relevance of automated CT-based body composition analyses in numerous medical contexts. With regard to sarcopenia and obesity, the determination of standardized cut-off values remains a key objective for future research in the field of automated body composition analysis. Overall, the method provides a reliable, reproducible, and clinically practical alternative to conventional anthropometric diagnostics.

### Strength and limitations

One of the strengths of the study is the innovative approach by applying pre-trained deep learning models for the automated analysis of body composition using thoracic CT scans in the NAPKON-SUEP cohort. This enables an objective and standardized evaluation that avoids subjective assessments and ensures a high degree of reproducibility. Compared to manual measurement methods, this technique offers more precise measurement of muscle, fat and bone volume, which is particularly advantageous in clinical applications. Another key aspect of the study is the holistic view of body composition. While previous research often focused on BMI or individual cross-sectional images, a 3D-approach was used here.

Despite these strengths, the study has some limitations. One important point is that body composition was only measured at a single point in time. There is a lack of data on how muscle mass changes over the course of the disease. Long-term observation would be helpful to better understand the extent to which such changes influence prognosis. Another limitation of the study is that height and weight were not objectively measured but were obtained through patient self-report during anamnesis, which may introduce reporting bias or inaccuracy.

Furthermore, the study focuses on muscle volume without considering functional aspects. However, for the diagnosis of sarcopenia the measurement of muscle strength is essential. Supplementing the study with a measurement of handgrip strength or other functional tests could help to further substantiate the clinical relevance of the results.

Overall, additional studies with long-term follow-up data and measurement of muscle strength would be necessary to further substantiate these findings.

## Conclusion

In summary, the NAPKON-SUEP cohort examined for the first-time muscle and fat volume of COVID-19 patients aged over 60 years in relation to mortality using automated 3D CT thorax analysis. The results showed that sarcopenia has a stronger influence on survival than obesity. The early identification of patients with critical muscle mass could be a key factor in improving survival rates and shortening the course of the disease. In this context, automated CT-based muscle mass determination offers a promising, innovative diagnostic tool. Further studies are needed to validate its usefulness in clinical practice and to enable its implementation.

## Data Availability

No datasets were generated or analysed during the current study.

## Abbreviations

ARDS: Acute Respiratory Distress Syndrome
BIA: Bioelectrical Impedance Analysis
BMI: Body Mass Index
COPD: Chronic Obstructive Pulmonary Disease
CT: Computed Tomography
DXA: Dual-Energy X-ray Absorptiometry
ICUAW: ICU-Acquired Weakness
IMAT: Intramuscular Adipose Tissue
LMU: Ludwig Maximilian University
NAPKON: National Pandemic Cohort Network
OR: Odds Ratio
PCR: Polymerase Chain Reaction
SARS-CoV-2: Severe Acute Respiratory Syndrome Coronavirus 2
SAT: Subcutaneous Adipose Tissue
SHIP-AI: Smart Hospital Information Platform – Artificial Intelligence
SD: Standard deviation
SO: Sarcopenic Obesity
SPSS: Statistical Package for the Social Sciences
SUEP: cross-sectoral platform (in German Sektorenübergreifende Plattform)
T4, T12, L2, L3: Thoracic and Lumbar Vertebrae
TAT: total adipose tissue

## References

[1] Zanders L, Kny M, Hahn A, Schmidt S, Wundersitz S, Todiras M, et al. Sepsis induces Interleukin 6, gp130/JAK2/STAT3, and muscle wasting. J Cachexia Sarcopenia Muscle. 2022;13(1):713–27. 34821076 10.1002/jcsm.12867 PMC8818599

[2] Liu W, Hu C, Zhao S. Sarcopenia and mortality risk of patients with sepsis: A Meta-Analysis. Int J Clin Pract. 2022;2022:4974410. 35685536 10.1155/2022/4974410 PMC9159150

[3] Hrdy O, Vrbica K, Kovar M, Korbicka T, Stepanova R, Gal R. Incidence of muscle wasting in the critically ill: a prospective observational cohort study. Sci Rep. 2023;13(1):742. 36639540 10.1038/s41598-023-28071-8 PMC9839699

[4] Wang Y, Tan S, Yan Q, Gao Y. Sarcopenia and COVID-19 outcomes. Clin Interv Aging. 2023;18:359–73. 36923269 10.2147/CIA.S398386 PMC10010141

[5] Yakti FAZ, Abusalah L, Ganji V. Sarcopenia and mortality in critically ill COVID-19 patients. Life (Basel). 2023;14(1). 10.3390/life14010024 PMC10820280 38255640

[6] Radigan KA, Nicholson TT, Welch LC, Chi M, Amarelle L, Angulo M, et al. Influenza A virus infection induces muscle wasting via IL-6 regulation of the E3 ubiquitin ligase Atrogin-1. J Immunol. 2019;202(2):484–93. 30530483 10.4049/jimmunol.1701433 PMC6324970

[7] Altuna-Venegas S, Aliaga-Vega R, Maguiña JL, Parodi JF, Runzer-Colmenares FM. Risk of community-acquired pneumonia in older adults with sarcopenia of a hospital from Callao, Peru 2010–2015. Arch Gerontol Geriatr. 2019;82:100–5. 30739000 10.1016/j.archger.2019.01.008 PMC8842506

[8] Maeda K, Akagi J. Muscle mass loss is a potential predictor of 90-Day mortality in older adults with aspiration pneumonia. J Am Geriatr Soc. 2017;65(1):e18–22. 27858956 10.1111/jgs.14543

[9] Herridge MS, Cheung AM, Tansey CM, Matte-Martyn A, Diaz-Granados N, Al-Saidi F, et al. One-year outcomes in survivors of the acute respiratory distress syndrome. N Engl J Med. 2003;348(8):683–93. 12594312 10.1056/NEJMoa022450

[10] Dinglas VD, Aronson Friedman L, Colantuoni E, Mendez-Tellez PA, Shanholtz CB, Ciesla ND, et al. Muscle weakness and 5-Year survival in acute respiratory distress syndrome survivors. Crit Care Med. 2017;45(3):446–53. 28067712 10.1097/CCM.0000000000002208 PMC5315580

[11] Marino M, Scuderi F, Provenzano C, Bartoccioni E. Skeletal muscle cells: from local inflammatory response to active immunity. Gene Ther. 2011;18(2):109–16. 20927136 10.1038/gt.2010.124

[12] Afzali AM, Müntefering T, Wiendl H, Meuth SG, Ruck T. Skeletal muscle cells actively shape (auto)immune responses. Autoimmun Rev. 2018;17(5):518–29. 29526638 10.1016/j.autrev.2017.12.005

[13] Nelke C, Dziewas R, Minnerup J, Meuth SG, Ruck T. Skeletal muscle as potential central link between sarcopenia and immune senescence. EBioMedicine. 2019;49:381–8. 31662290 10.1016/j.ebiom.2019.10.034 PMC6945275

[14] Shoemaker ME, Salmon OF, Smith CM, Duarte-Gardea MO, Cramer JT. Influences of vitamin D and Iron status on skeletal muscle health: A narrative review. Nutrients. 2022;14(13). 10.3390/nu14132717 PMC9268405 35807896

[15] Pecora F, Persico F, Argentiero A, Neglia C, Esposito S. The role of micronutrients in support of the immune response against viral infections. Nutrients. 2020;12(10). 10.3390/nu12103198 PMC7589163 33092041

[16] de Leeuw AJM, Oude Luttikhuis MAM, Wellen AC, Müller C, Calkhoven CF. Obesity and its impact on COVID-19. J Mol Med (Berl). 2021;99(7):899–915. 33824998 10.1007/s00109-021-02072-4 PMC8023779

[17] Papadimitriou-Olivgeris M, Aretha D, Zotou A, Koutsileou K, Zbouki A, Lefkaditi A, et al. The role of obesity in Sepsis outcome among critically ill patients: A retrospective cohort analysis. Biomed Res Int. 2016;2016:5941279. 27777948 10.1155/2016/5941279 PMC5061945

[18] Molfino A, Imbimbo G, Rizzo V, Muscaritoli M, Alampi D. The link between nutritional status and outcomes in COVID-19 patients in ICU: is obesity or sarcopenia the real problem? Eur J Intern Med. 2021;91:93–5. 34246503 10.1016/j.ejim.2021.06.028 PMC8249749

[19] Marra M, Sammarco R, De Lorenzo A, Iellamo F, Siervo M, Pietrobelli A, et al. Assessment of body composition in health and disease using bioelectrical impedance analysis (BIA) and dual energy X-Ray absorptiometry (DXA): A critical overview. Contrast Media Mol Imaging. 2019;2019:3548284. 31275083 10.1155/2019/3548284 PMC6560329

[20] Pickhardt PJ, Summers RM, Garrett JW. Automated CT-Based body composition analysis: A golden opportunity. Korean J Radiol. 2021;22(12):1934–7. 34719894 10.3348/kjr.2021.0775 PMC8628162

[21] Schons M, Pilgram L, Reese JP, Stecher M, Anton G, Appel KS, et al. The German National pandemic cohort network (NAPKON): rationale, study design and baseline characteristics. Eur J Epidemiol. 2022;37(8):849–70. 35904671 10.1007/s10654-022-00896-z PMC9336157

[22] Hosch R, Kattner S, Berger MM, Brenner T, Haubold J, Kleesiek J, et al. Biomarkers extracted by fully automated body composition analysis from chest CT correlate with SARS-CoV-2 outcome severity. Sci Rep. 2022;12(1):16411. 36180519 10.1038/s41598-022-20419-w PMC9524347

[23] Koitka S, Kroll L, Malamutmann E, Oezcelik A, Nensa F. Fully automated body composition analysis in routine CT imaging using 3D semantic segmentation convolutional neural networks. Eur Radiol. 2021;31(4):1795–804. 32945971 10.1007/s00330-020-07147-3 PMC7979624

[24] Kroll L, Mathew A, Baldini G, Hosch R, Koitka S, Kleesiek J, et al. CT-derived body composition analysis could possibly replace DXA and BIA to monitor NET-patients. Sci Rep. 2022;12(1):13419. 35927564 10.1038/s41598-022-17611-3 PMC9352897

[25] Obesity. Preventing and managing the global epidemic. Report of a WHO consultation. World Health Organ Tech Rep Ser. 2000;894:i–xii. 11234459

[26] Cruz-Jentoft AJ, Bahat G, Bauer J, Boirie Y, Bruyère O, Cederholm T, et al. Sarcopenia: revised European consensus on definition and diagnosis. Age Ageing. 2019;48(1):16–31. 30312372 10.1093/ageing/afy169 PMC6322506

[27] Donini LM, Busetto L, Bischoff SC, Cederholm T, Ballesteros-Pomar MD, Batsis JA, et al. Definition and diagnostic criteria for sarcopenic obesity: ESPEN and EASO consensus statement. Obes Facts. 2022;15(3):321–35. 35196654 10.1159/000521241 PMC9210010

[28] Schiaffino S, Albano D, Cozzi A, Messina C, Arioli R, Bnà C, et al. CT-derived chest muscle metrics for outcome prediction in patients with COVID-19. Radiology. 2021;300(2):E328–36. 33724065 10.1148/radiol.2021204141 PMC7971428

[29] Liu J, Ye Z, Xiang J, Wang Q, Zhao W, Qin W, et al. Association of muscle mass and radiodensity assessed by chest CT with all-cause and cardiovascular mortality in Hemodialysis patients. Int Urol Nephrol. 2024;56(11):3627–38. 38865001 10.1007/s11255-024-04113-6

[30] Sumbal R, Sumbal A, Ali Baig MM. Which vertebral level should be used to calculate sarcopenia in covid-19 patients? A systematic review and meta-analysis. Clin Nutr ESPEN. 2023;56:1–8. 37344057 10.1016/j.clnesp.2023.04.022 PMC10159940

[31] Schinas G, Dimakopoulou V, Dionysopoulos K, Fezoulidi G, Vlychou M, Vassiou K, et al. Radiologic features of T10 paravertebral muscle sarcopenia: prognostic factors in COVID-19. J Clin Med Res. 2023;15(7):368–76. 37575354 10.14740/jocmr4963 PMC10416190

[32] Ying-Hao P, Hai-Dong Z, Yuan F, Yong-Kang L, Sen L, Wei-Long X, et al. Correlation of CT-derived pectoralis muscle status and COVID-19 induced lung injury in elderly patients. BMC Med Imaging. 2022;22(1):144. 35962312 10.1186/s12880-022-00872-9 PMC9372984

[33] Palmisano A, Gnasso C, Cereda A, Vignale D, Leone R, Nicoletti V, et al. Chest CT opportunistic biomarkers for phenotyping high-risk COVID-19 patients: a retrospective multicentre study. Eur Radiol. 2023;33(11):7756–68. 37166497 10.1007/s00330-023-09702-0 PMC10173240

[34] Ulugerger Avci G, Bektan Kanat B, Can G, Suzan V, Unal D, Degirmenci P, et al. The impact of sarcopenia and obesity on mortality of older adults: five years results. Ir J Med Sci. 2023;192(5):2209–16. 37202585 10.1007/s11845-023-03392-9

[35] Pishgar F, Shabani M, Quinaglia ACST, Bluemke DA, Budoff M, Barr RG, et al. Quantitative analysis of adipose depots by using chest CT and associations with All-Cause mortality in chronic obstructive pulmonary disease: longitudinal analysis from mesarthritis ancillary study. Radiology. 2021;299(3):703–11. 33825508 10.1148/radiol.2021203959 PMC8165946

[36] Addison O, Marcus RL, Lastayo PC, Ryan AS. Intermuscular fat: a review of the consequences and causes. Int J Endocrinol. 2014;2014:309570. 24527032 10.1155/2014/309570 PMC3910392

[37] Schluessel S, Mueller B, Tausendfreund O, Rippl M, Deissler L, et al. CT-derived muscle volumes as a surrogate for DXA in sarcopenia diagnosis: a 3D Deep Learning Approach. 2025, under revision.

[38] Huang YT, Tsai YS, Lin PC, Yeh YM, Hsu YT, Wu PY, et al. The value of artificial Intelligence-Assisted imaging in identifying diagnostic markers of sarcopenia in patients with Cancer. Dis Markers. 2022;2022:1819841. 35392497 10.1155/2022/1819841 PMC8983171

[39] Gao YD, Ding M, Dong X, Zhang JJ, Kursat Azkur A, Azkur D, et al. Risk factors for severe and critically ill COVID-19 patients: A review. Allergy. 2021;76(2):428–55. 33185910 10.1111/all.14657

[40] Kastora S, Patel M, Carter B, Delibegovic M, Myint PK. Impact of diabetes on COVID-19 mortality and hospital outcomes from a global perspective: an umbrella systematic review and meta-analysis. Endocrinol Diabetes Metab. 2022;5(3):e00338. 35441801 10.1002/edm2.338 PMC9094465

[41] Esposito AJ, Menon AA, Ghosh AJ, Putman RK, Fredenburgh LE, El-Chemaly SY, et al. Increased odds of death for patients with interstitial lung disease and COVID-19: A Case-Control study. Am J Respir Crit Care Med. 2020;202(12):1710–3. 32897754 10.1164/rccm.202006-2441LE PMC7737588

[42] Menon T, Gandhi SAQ, Tariq W, Sharma R, Sardar S, Arshad AM, et al. Impact of chronic kidney disease on severity and mortality in COVID-19 patients: A systematic review and Meta-analysis. Cureus. 2021;13(4):e14279. 33959457 10.7759/cureus.14279 PMC8093366

[43] Cho MR, Lee S, Song SK. A review of sarcopenia pathophysiology, diagnosis, treatment and future direction. J Korean Med Sci. 2022;37(18):e146. 35535373 10.3346/jkms.2022.37.e146 PMC9091430

[44] García-Pérez-de-Sevilla G, Sánchez-Pinto Pinto B. Effectiveness of physical exercise and neuromuscular electrical stimulation interventions for preventing and treating intensive care unit-acquired weakness: A systematic review of randomized controlled trials. Intensive Crit Care Nurs. 2023;74:103333. 36283894 10.1016/j.iccn.2022.103333

[45] Vorona S, Sabatini U, Al-Maqbali S, Bertoni M, Dres M, Bissett B, et al. Inspiratory muscle rehabilitation in critically ill adults. A systematic review and Meta-Analysis. Ann Am Thorac Soc. 2018;15(6):735–44. 29584447 10.1513/AnnalsATS.201712-961OC PMC6137679

[46] Nagano A, Wakabayashi H, Maeda K, Kokura Y, Miyazaki S, Mori T, et al. Respiratory sarcopenia and sarcopenic respiratory disability: concepts, diagnosis, and treatment. J Nutr Health Aging. 2021;25(4):507–15. 33786569 10.1007/s12603-021-1587-5 PMC7799157

[47] Pu L, Ashraf SF, Gezer NS, Ocak I, Dresser DE, Leader JK, et al. Estimating 3-D whole-body composition from a chest CT scan. Med Phys. 2022;49(11):7108–17. 35737963 10.1002/mp.15821 PMC10084085

[48] Salhöfer L, Bonella F, Meetschen M, Umutlu L, Forsting M, Schaarschmidt BM et al. Automated 3D-Body composition analysis as a predictor of survival in patients with idiopathic pulmonary fibrosis. J Thorac Imaging. 2025;40(2). 10.1097/RTI.0000000000000803 PMC11837968 39183570

